# The Development of an Electronic Medical Record System to Improve Quality of Care for Individuals With Type 1 Diabetes in Rwanda: Qualitative Study

**DOI:** 10.2196/52271

**Published:** 2024-09-20

**Authors:** Nathalie Bille, Dirk Lund Christensen, Stine Byberg, Michael Calopietro, Crispin Gishoma, Sarah Fredsted Villadsen

**Affiliations:** 1 Section of Global Health Department of Public Health University of Copenhagen Copenhagen K Denmark; 2 Department of Digital Health Solutions World Diabetes Foundation Bagsværd Denmark; 3 Department of Clinical Epidemiology Clinical Research, Copenhagen University Hospital Steno Diabetes Center Copenhagen Herlev Denmark; 4 Rwanda Diabetes Association Kigali Rwanda; 5 Section of Social Medicine Department of Public Health University of Copenhagen Copenhagen Denmark

**Keywords:** type 1 diabetes, electronic medical record systems, Rwanda, complex interventions, intervention development, diabetes, diabetic, DM, diabetes mellitus, chronic disease, chronic diseases, qualitative, focus group, quality of care, clinical outcome, clinical outcomes, public health interventions, public health intervention, design, electronic health record, electronic health records, EHR, medical records, medical record

## Abstract

**Background:**

Electronic medical record (EMR) systems have the potential to improve the quality of care and clinical outcomes for individuals with chronic and complex diseases. However, studies on the development and use of EMR systems for type 1 (T1) diabetes management in sub-Saharan Africa are few.

**Objective:**

The aim of this study is to analyze the need for improvements in the care processes that can be facilitated by an EMR system and to develop an EMR system for increasing quality of care and clinical outcomes for individuals with T1 diabetes in Rwanda.

**Methods:**

A qualitative, cocreative, and multidisciplinary approach involving local stakeholders, guided by the framework for complex public health interventions, was applied. Participant observation and the patient’s personal experiences were used as case studies to understand the clinical care context. A focus group discussion and workshops were conducted to define the features and content of an EMR. The data were analyzed using thematic analysis.

**Results:**

The identified themes related to feature requirements were (1) ease of use, (2) automatic report preparation, (3) clinical decision support tool, (4) data validity, (5) patient follow-up, (6) data protection, and (7) training. The identified themes related to content requirements were (1) treatment regimen, (2) mental health, and (3) socioeconomic and demographic conditions. A theory of change was developed based on the defined feature and content requirements to demonstrate how these requirements could strengthen the quality of care and improve clinical outcomes for people with T1 diabetes.

**Conclusions:**

The EMR system, including its functionalities and content, can be developed through an inclusive and cocreative process, which improves the design phase of the EMR. The development process of the EMR system is replicable, but the solution needs to be customized to the local context.

## Introduction

Electronic medical record (EMR) systems are electronic platforms that contain individual health records for patients, maintained by health care professionals (HCPs) and health care organizations. EMRs may include patients’ medical history, diagnoses, clinical test results, and treatment plans [1]. Studies from high-income countries (HICs) have shown that the use of EMR systems has the potential to identify and target high-risk patients, autogenerate appointments, and send reminders to patients who do not show up for consultation. Moreover, other potential benefits of the use of EMR include better organization of relevant data and providing HCPs with feedback on their care for individuals with diabetes [2-4]. These features can lead to significant improvements in care and outcomes, as supplies, adjustments to medicines and timely referral can be better ensured [3,5]. Little evidence is available on the development, use, and effects of EMR systems for life-long chronic disease management, such as type 1 diabetes (T1 diabetes), in sub-Saharan Africa [6]. To increase the likelihood of successful implementation, it is crucial to know existing practices and the structure of the current system in order to know what changes are needed and feasible under the local circumstances [7]. However, studies within other disease areas, such as HIV, have shown positive effects on quality of care using EMR systems [8,9]. A study from Kenya showed that the implementation of an EMR system was significantly associated with receiving antiretroviral therapy among patients with HIV and having at least one CD4 test done compared to that before the intervention using paper records [10,11].

Health care systems, access to health care, and health care–seeking behavior differ between countries. Requirements, priorities, and local constraints related to EMR systems in low- and middle-income countries (LMICs) are not well understood [12]. To understand the local context for the people and the health care system involved in the intervention, social practices, needs, possibilities, and potential challenges should be taken into consideration [13]. In this study, we aimed to explore the requirements and needs for improvement in the care processes. This can be facilitated by an EMR system, and these can inform the development of an EMR system for individuals with T1 diabetes in Rwanda. Therefore, the purpose of the design stage was primarily focused on (1) understanding the clinical care context and workflows, (2) defining functional requirements, and (3) defining content requirements.

## Methods

### Study Participants

The recruitment of study participants was based on a user-centered design approach and therefore included people with (1) significant understanding of the T1 diabetes care pathway and delivery of care, (2) deep experience of receiving T1 diabetes care, and (3) expert knowledge of national digital health strategies or expertise in the design and development of electronic health record platforms (Table 1). Participants were therefore selected to reflect a range of specific characteristics and experiences known to affect the experience and delivery of health care, including gender, years of experience, practitioner role, and diverse patient socioeconomic characteristics. Initially, we identified international expert stakeholders within T1 diabetes and EMR systems (Textbox 1), and meetings were held for inspirational and insight purposes. Thereafter, we identified relevant local stakeholders and conducted the primary data collection for the EMR development (elaborated below).

**Table 1:** Study participant types in different research activities.

			Focus group discussion, n=10		Workshops, n=8		Patient interview, n=13
**Participant type**							
	Caregiver (nurses and endocrinologist)^a^	7		3		—	
	Project manager^a^	2		2		—	
	T1 diabetes individuals^b^	—		—		13	
	IT expert/software developer^c^	1		2		—	
	Policy makers^c^	—		1		—	
**Sex**							
	Female	3		1		7	
	Male	7		7		6	

^a^Significant understanding of the T1 diabetes care pathway and delivery of care.

^b^Deep experience of receiving T1 diabetes care.

^c^Expert knowledge of national digital health strategies or in the design and development of electronic health record platforms.

**International expert stakeholders within T1 diabetes and electronic medical record (EMR) systems.**

**SWEET**: An international network for paediatric diabetes centers, established in 2008, striving to improve treatment outcomes using standardized documentation and objective comparison of quality indicators.

**Life for a Child (LFAC):** An NGO established in 2000, providing young people in under-resourced countries with life-saving insulin and supplies and has the vision that no child should die of diabetes.

**Changing Diabetes in Children (CDIC)**: A public-private partnership established in 2009. The partnership provides comprehensive care for children and young people living with type 1 diabetes in low- and middle-income countries.

**International Research Institute (IRT)**: An independent, non-profit institute established in 1958 that provides research, development, and technical services to government and commercial clients. RTI’s mission is to improve the human condition by turning knowledge into practice.

**World Diabetes Foundation (WDF):** An independent foundation founded in 2002 by Novo Nordisk A/S, and is today a leading global funder of diabetes prevention and care projects in low- and middle-income countries.

### Data Collection

Researchers with training in qualitative research collected data via 4 activities: technical requirements focus group discussions (FGDs), clinical and contextual observations, patient interviews, and user design workshops. All activities were conducted in English, except for the patient interviews, which were conducted in the local language [Kinyarwanda] and subsequently translated to English by a project manager. The primary data collection was conducted in November 2021 (1 month), mid-February to mid-March 2022 (1 month), and June 2022 (1 month). The different study participant types across research activities are summarized in Table 1.

#### Semistructured Focus Group Discussion

The purpose of the semistructured FGD was to get a shared understanding of challenges related to current data collection practices and patient monitoring, as well as the needs and requirements for a new electronic system on how to improve follow-up and care for individuals with T1 diabetes. The participants were asked to talk about what data elements and functionalities they would like to see in a new EMR system. The group discussion was chosen to allow a more informal environment where participants could inspire and supplement each other. The participants included nurses (n=7) working at the Rwanda Diabetes Association (RDA), who were familiar with current practices and were expected to use the new EMR system. In addition to this, a few other participants with different professional backgrounds (n=3) were included to get a full understanding of the requirements for the system and contextual insights. The FGD was held after a scheduled meeting at RDA. All participants were either recruited in person at the RDA office or by phone call, and attendance was voluntary. All participants gave signed informed consent prior to the interview. A semi-structured interview guide was used, the session was audio recorded, and an assistant observer was present to take notes during the 2.5-hour interview session. An overweight group of nurses from RDA, representatives of both genders and with a representative age range of 24-54, mean 38.9 (SD 10), were recruited.

#### Workshops

The workshops served to make room for interaction and discussions about the EMR system requirements among a multidisciplinary group of people. Participants from all 3 stakeholder groups (Table 1) were invited to a joint meeting. Furthermore, 4 physical meetings and 4 web-based sessions with the software developer of the EMR system, a pediatrician from Rwanda Military Hospital, the management team from RDA, and the primary investigator (NB) from WDF were conducted. At the workshops, the software developer presented demo versions of the new software, and the features were discussed. Minutes from all meetings were recorded by NB and distributed for approval. From meeting to meeting, changes were suggested, and subsequently, the EMR system was updated.

#### Participant Observation and Patient Stories

Participant observations were conducted by the primary investigator (NB) at the RDA clinic in Kigali and at 2 district hospitals; Nemba Hospital and Rumera-Rukoma Hospital. The observations served to better understand the context, the workflow of the HCPs and the RDA staff, current diabetes care processes, and health information collection and recording. In addition to participant observations, patient stories were also collected at the clinic visits. During the clinic visits, 10 individuals with T1 diabetes were informally interviewed (between 15 and 35 min). In addition, 3 individuals were invited by the project manager at RDA to tell their stories about how it is to live with T1 diabetes in Rwanda, and what challenges they face in seeking care and managing their disease.

### Analytical Approach

The analysis was inspired by a reflexive thematic approach, focusing on identifying patterned meanings (themes) in data sets [14] related to features and contents. Data triangulation methodology was applied to get a more comprehensive understanding and to enhance the validity of the analyses through the convergence of information from the different data sources and methodologies [15].

The research methodology was guided by the MRC framework related to complex intervention development [16,17]. The approach was partnership-driven but also used a theory of change (ToC) model [17,18]. A ToC model articulates how an intervention is expected to generate outcomes [19], thus, in our case, how the EMR system is expected to link to expected outcomes. Features and content to be incorporated in the EMR system were identified. “Features” encompasses all the functionalities of the system and “content” encompasses all the information and data elements of importance to be included in the EMR system to help the monitoring and care of T1 diabetes individuals. The needs and requirements were combined with local clinical guidelines based on international standards for T1 diabetes care.

### Ethical Considerations

The project was approved by the Institutional Review Board, College of Medicine and Health Sciences, University of Rwanda (FWA assurance number 0001971, IRB 00001497 of IORG 0001100). Informed consent forms were signed for all participants. The consent forms emphasized that participation was entirely voluntary and all data would be kept anonymous. Participants were free to withdraw from the study at any time without further explanation. No compensations for participation were given.

## Results

### Understanding the Clinical Care Context and Workflows

Since 2009, RDA has been the main health care provider for T1 diabetes in Rwanda, conducting continuous follow-up on children and youth with T1 diabetes for treatment and control purposes. The RDA operates according to national diagnosis and treatment guidelines. Individuals are typically diagnosed with T1 diabetes at the local health facility, the district hospital, a private clinic, or the RDA clinic upon presentation with symptoms of T1 diabetes. Diagnostic confirmation is usually based on glycated hemoglobin (HbA_1c_) testing, age at diagnosis, response to insulin, presence of diabetic ketoacidosis symptoms, and underweight [20-22].

**Figure 1:**
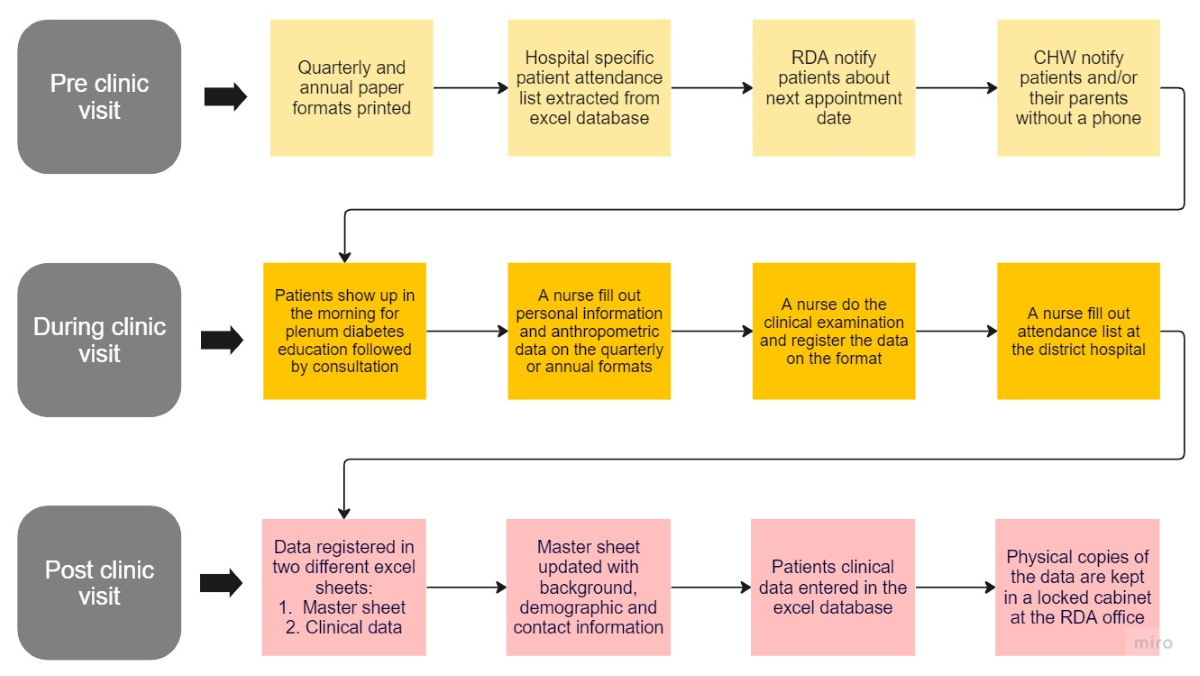
Follow-up and data collection flow. CHW: Community Health Worker; RDA: Rwanda Diabetes Association.

The RDA has 1 clinic in the capital, Kigali, and works closely with the noncommunicable disease clinics at the district hospitals located throughout the country. Individuals with T1 diabetes living in the Kigali region go directly to the RDA clinic. For individuals with T1 diabetes living outside of Kigali, a team of diabetes nurses and educators from RDA conduct quarterly and annual visits to the district hospitals, providing treatment and care [22,23]. The annual visit is an extensive version of the health examination done at the quarterly visits. Within approximately 3 weeks of the scheduled visit, individuals with T1 diabetes or their legal guardians are notified about the next appointment date, either directly by RDA via phone call, by a nurse from the district hospital, or by the community health worker within the community. On the day of the appointment, T1 diabetes individuals show up in the morning at 9:00 AM for plenum diabetes education, followed by individual examinations conducted by RDA HCPs. All data are collected on paper forms, which are subsequently registered in an Excel spreadsheet database. Transfer of data from paper to spreadsheet is typically carried out at the end of the day, when the nurses have a nightshift or when they have some time left between other tasks. The physical copies are stored in a locked cabinet at the RDA office in Kigali. The follow-up and data collection flow are summarized in Figure 1.

### Feature Requirements

The following themes related to the concept of features were identified: ease of use (user-friendly), reporting (automatic report generation), clinical decision support tool (regulation), data validity (data quality), patient follow-up, and data protection and training.

#### Design: Ease of Use

The structure, design, and interface of the system were identified as important aspects of the new EMR system. It must be user-friendly, intuitive, and developed with a focus on the user (RDA staff). The HCP expressed that the structures and flows of how, and when, the different information should be obtained needed to be considered, and the data elements should be grouped in a meaningful way, as expressed by one of the project managers:

It should be considered how we group and organise the order of the data being collected to improve efficiency. The information collected in the system should match the workflow of RDA.

Project manager 1 (FGD)

Participants suggested that patient demographics are only captured one time and recorded in the registration form. Further to this, during the quarterly visit, the most important variables (vitals and results of medical tests) should be captured. Additional information related to patient characteristics that change over time (eg, lifestyle, sociodemographic factors, mental health, etc) and results of microvascular and macrovascular screenings. A balance between “need to know” and “nice to know” should be carefully considered, taking the limited time for consultation per person into account. The less time HCPs spend collecting and registering the data, the more time they have to do more tests, ask more questions, and educate the individual about their disease. Therefore, fewer clicks and time optimizing functions were brought up as important factors, as one project manager explained:

The system should have few clicks to ease the data collections processes. The system should not be too complicated to use and therefore the design and structure of the system are important.

Project manager 1 (FGD)

#### Automatic Report Generation

The new EMR could simplify the time-consuming task of generating frequent and routine reports. In the quote below, one manager explains the tedious work of preparing the report:

Every month RDA is obligated to upload a report with the status of the T1 diabetes individuals to the MoH. It can take 3-4 days preparing the report. It could be very helpful if the system can generate the data for the reports, including numbers of individuals with newly identified T1 diabetes.

Project manager 2 (workshop)

The time gained by using web-based analysis tools for report preparation and outcome monitoring can be used for other important or patient-related tasks. The reports generated by the system should include summary statistics, including graphs and figures giving an overview of incidence and prevalence rates and the health status of the T1 diabetes population. This was expressed by one project manager:

It will be useful if the system can provide basic statistics, so it is easier to compare patients across districts and between sex to better target actions and improve care for those who need it most… The reports can be very useful for the management team for control and target purposes.

Project manager 1 (FGD)

#### Clinician Decision Support Tool (Regulation)

The system can generate aggregated reports, which can help track patient outcomes, support clinical management, and improve clinical efficiency. The system can assist in prompt and effective patient management. At point-of-care, the RDA staff can view a summary of the medical records of the individuals with T1 diabetes, showing a chart with clinical and anthropometric measures.

Reporting an overview of HbA1c levels, blood glucose levels, height, weight, BMI and diastolic and systolic blood pressure is important to see if the patients improve their glycaemic control and in other relevant factors over time.

Project manager 2 (workshop)

The HCPs can track the patient’s development in disease management over time as well as identify critical values using real-time data. When key screening tests for comprehensive diabetes care are due or clinical test results are outside the normal range, the RDA staff will receive alerts to facilitate clinical decision-making and considerations for therapeutic interventions. The HCPs can change the treatment regimen according to the recorded blood glucose and HbA_1c_ levels if the values are critically high or low. Not only can the system help provide timely screening and clinical tests, but it can also help avoid unnecessary examinations, saving time and resources. One nurse mentioned how care can be improved and ultimately optimized by providing the right examination in a timely manner:

The system should be able to remind the HCPs when it is time for which tests, ensure timely examinations and avoid repeating exams that is not necessary. The system could also alert about critical test results to help the nurses optimizing care.

Nurse 1 (FGD)

Using the system and the collected data to guide the HCPs in improving care for individuals with T1 diabetes was a shared belief among the nurses and project managers participating in the FGD.

#### Data Validity

Since clinical decision-making is based on the data entered and stored in the system, it is critical that the data be as reliable as possible. The EMR system should incorporate multiple functions, helping the RDA staff reduce errors and limit missing information to improve the quality of the data. These include missing data checks, data value checks, and data logic checks. The system should help ensure that meaningless values are avoided, as expressed by a project manager:

The system can help reduce human errors. If there are restrictions on some information, it can improve the data. The Rwandan phone numbers have 9 digits—registering more or less than 9 digits should not be allowed by the system.

Project manager 1 (FGD)

This does not mean that “free text” boxes must be excluded, as not all data fit into normal and predefined categories or patterns. In the new system, some data elements require further description, necessitating the inclusion of “free text” boxes. Additionally, information boxes or subtitles should be included for specific data elements to provide clarifying information to the HCPs. For example, how a family size is defined, and the difference between prescribed and self-reported insulin dosage.

#### Patient Follow-Up

The EMR system should record scheduled visits and send SMS reminders to patients in the local language [Kinyarwanda] reminding them of the date and time of their next visit. This feature is necessary to improve clinic-visit attendance, which can be a challenge for people with T1 diabetes. If a patient does not attend a scheduled appointment, the EMR system should generate a notification so that additional action can be taken to find the patient and reschedule a new appointment. Tracking the number of coherent missed appointments can also help target efforts to find the patients. After several workshops, the clinicians agreed that lost follow-up should be considered after 3 consecutive missed appointments or no-shows for more than a year. This is an arbitrary definition, so it was also agreed that the threshold could be revised after an implementation evaluation. Moreover, it is important to register individuals who have passed away so that follow-up efforts are only targeted at those still alive. The system should also track migration history, easing the process of locating the right individuals. Reliable patient identification can be ensured by autogenerating unique IDs that refer to a numerical code for a specific patient. This feature should help support deduplication and avoid 2 individuals having the same ID, as this confuses the identification of patients. The issue was observed from participant observations in the field:

Sometimes when identifying people in the excel database one person was registered more than once with two different ID numbers. Also, in other occasions we found out that two different people has been given the same ID, meaning that the data was mixed.

Participant observation

Other relevant, unique national IDs issued to citizens of Rwanda should also be stored to help identify patients and reduce duplication of patient records.

#### Data Protection

In Rwanda, internet and electricity are available in most places throughout the country, but their quality varies. Connectivity failure can lead to data loss if offline functioning is not in place. Therefore, the EMR system should be able to capture data offline and automatically upload it to a server or cloud-based function after re-connecting to the internet. This concern was raised by several of the nurses:

The system cannot work without internet. Sometimes the internet fails at the countryside and in the rural areas, why it would be better if the system can work offline. The system can only be sustainable if the system works offline- offline functioning ensures that no data is lost.

Nurse 2 (FGD

However, this was also discussed with a project manager and the IT expert at one of the workshops, where it was mentioned that internet connectivity rarely fails and is therefore less of a concern. It was further expressed as follows:

We needed to consider the pros and cons of including an offline functionality in the system because it might negatively impact the possibility of making adaption and changes in the system as we go into implementation phase.

Software developer (workshop)

Moreover, the patient’s data should be protected and respected, and therefore the system should comply with Rwanda’s National Data Protection law [24]. Patient information should only be released and used by others with the patient’s permission, with written informed consent. The data should be secured by giving authorized access to the system with personalized usernames and passwords. Users of the system should have different authorizations depending on their functions, responsibilities, and pre-established role-based privileges. Allowing more HCPs to have access to the data will require additional governmental approvals.

### Content Requirements

The following themes related to the concept of content were identified: treatment regimen, complications, mental health, and socioeconomic conditions.

#### Treatment Regimen

The treatment regimen was mentioned as a complex matter in the care of T1 diabetes, as insulin intake and treatment plans have to be individualized according to the lifestyle and general condition. The RDA staff that participated in the workshop expressed the needs that must be carefully considered when providing care:

We experience that many patients have high blood glucose levels and we need to do something about it. The insulin dosage needs to be adjusted according to the blood sugar levels - we need to control and take actions and find the right treatment… also we need to consider the diet, physical activity and other factors that might impact the blood sugar levels.

Project manager 2 (workshop)

A lot of different factors might have an influence on how the individual is managing the disease. Even though insulin is a necessity for survival, it is not the only factor contributing to good diabetes management. Many individuals are still in poor control due to other factors than insulin availability and affordability. It is important to adjust and refine the insulin dosage and injections according to the time of day, insulin types, food intake, physical activity level, and insulin storage, as it impacts the effect if it is exposed to high temperatures. It is also crucial that the individual continuously monitors blood glucose levels if self-adjustment of insulin dosage is needed. Sometimes individuals with T1 diabetes did not have access to glucometers for various reasons; the glucometer was lost, out of batteries, or defective.

#### Complications

Information about self-reported cases of acute complications, such as diabetic ketoacidosis, hypoglycemia, and acute hospitalization, as well as information related to chronic complications, should be obtained and registered in the system. This includes screening, diagnosing, and referring to treatment for nephropathy (microalbuminuria), retinopathy, and neuropathy. One nurse mentioned:

We need to screen the patients for complications to ensure timely referral and treatment... It is critical to investigate if chronic complications have developed so we can either prevent them from occur or take it into account in the care we provide.

Nurse 1(FGD)

#### Mental Health

Mental health problems were mentioned to be an overlooked but serious consequence of T1 diabetes in Rwanda. Individuals are usually missed or underdiagnosed with depression. Thus, additional efforts are needed to ensure a screening process for mental health issues in people living with T1 diabetes. A standardized screening tool for identifying diabetes-related distress should be incorporated into the system with the balance of being able to detect those with mental health issues and not overloading the HCPs with too much additional work. The tool should help guide referrals to a mental health specialist.

#### Socioeconomic Conditions

Finally, socioeconomic and demographic information (eg, educational level, job status, insurance, and marital status) were considered important—but particularly whether or not the patient has support from family or friends to manage the treatment regimen.

### Theory of Change

**Figure 2:**
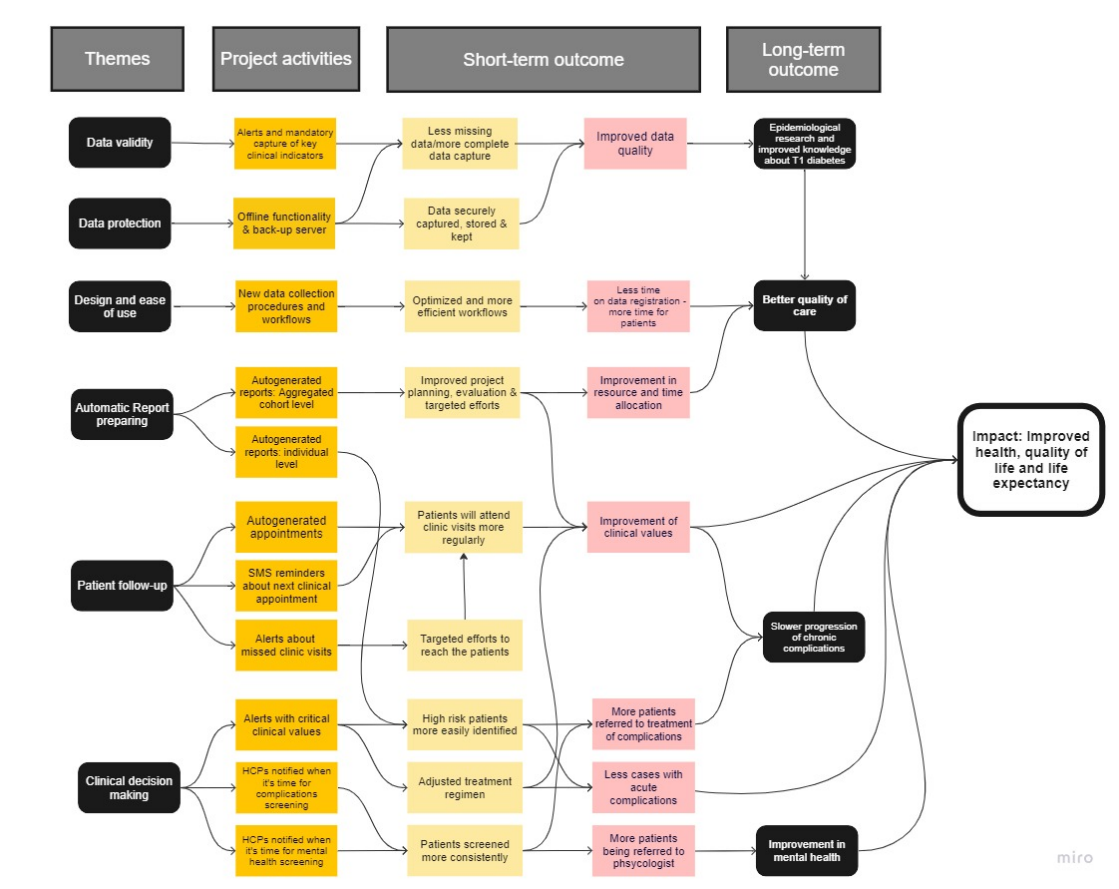
Theory of change—a set of assumptions about the relationship between themes, project activities, and expected short- and long-term outcomes. HCP: health care professional.

The findings from the thematic analysis were used to generate a ToC. The EMR system and its features as well as the data collected are expected to make a change. Figure 2 summarizes how the different themes identified in the analyses relate to project activities, expected output, and outcomes.

## Discussion

### Summary of Main Findings

The features and content of the EMR system were discussed from the perspective of improving clinical outcomes for individuals with T1 diabetes in Rwanda. Improving clinical efficiency through a new EMR system could reduce the time spent on data registration, leaving more time for consultations with individuals with T1 diabetes. Automatic report generation could also reduce time and better inform HCPs and policy makers about the status of T1 diabetes care in Rwanda. This can be used to guide clinical practice, monitor clinical quality, guide service delivery planning, and allocate resources. Longitudinal data collection at the individual level should be used for tracking patients over time and supporting clinical decision-making. This is in addition to using real-time data to guide the treatment regimen and disease management, all of which can lead to improved clinical efficiency, quality of care, and better outcomes. A fundamental part of improving clinical decision-making and follow-up on individuals with T1 diabetes is the quality of the data. This ensures that the right people are timely identified and that treatment regimens are based on valid information. Moreover, it is critical to capture, store, and ensure good data protection, as well as to comply with national regulations and laws to keep the data safe and confidential. It requires offline functionality in case of internet connectivity failure and a server system to ensure back-up of the data. Finally, it is important to collect the right information (content). In addition to the clinical data, information about treatment regimen, socioeconomic condition, screening for complications, and mental health status were identified as being of great importance to improving the care and clinical outcomes for individuals with T1 diabetes.

### Comparison with Prior Work

As research on EMR systems for T1 diabetes in low-income countries has been very limited, we discussed our findings with studies in HICs or other disease areas. In a review by Ali Mohammad et al [25], the enhanced functionalities integrated in the EMR systems were defined as reminders, management prompts, self-care support, provider feedback, and patient report generation. These functionalities are similar to most of the features identified as important in this study: SMS reminders for consultation, reminders for complications screening, management prompts related to critical clinical values, and missed appointments. This was in addition to better self-care support guided by data-informed treatment regimen and autogenerated reports on an aggregated and individual level. Importantly, further discussions are needed on how these standardized values are interpreted in combination with clinical decision-making. Strong clinical competence and not only technical skills are needed to navigate the EMR system without giving too much influence to the individual indicators.

We found that offline functionality was viewed as an important feature related to data protection and ensuring that data are securely captured and uploaded to a backup server. In contrast, the benefits of using a networking EMR system were mentioned in the study by Fraser et al [12]. It was stated that internet access allows a more flexible design, data can be more easily shared at multiple sites, and multiple users can enter the data simultaneously. The study mainly included EMR systems from HICs, where internet failure is considered less of a problem. However, another study reviewing the use of EMR systems in sub-Saharan Africa in other disease areas reported that poor network structure can be a barrier to the adaptation of the EMR systems [8].

Lack of comfort among HCPs to use an EMR system has also been identified as a potential barrier, which can underscore the importance of an easy-to-use system and training of users to increase adaption to the EMR system. In the review by Fraser et al [12], none of the mentioned systems were designed for T1 diabetes, but aligned with the findings of this study, ease of use and training were mentioned as critical factors for a successful implementation. The system should capture the minimum data necessary for the task, and data items should be structured to simplify data entry and optimize use.

In this study, treatment regimen, mental health, and sociodemographic conditions were highlighted as themes of importance for good diabetes management. Khater et al [26] state that living with T1 diabetes, especially in LMICs, can feel overwhelming for both children and parents because constant vigilance and complex and demanding treatment regimen are required for proper care with inadequate resources. It was also found that depression in children and adolescents with T1 diabetes has been associated with suboptimal glycemic control and an increased risk of developing complications and recurrent diabetic ketoacidosis [26]. Therefore, information related to this is critical and aligned with the findings of this study. Screening for diabetes-related distress can help refer patients to a psychologist to get better coping strategies about how to live better mentally with the disease. Moreover, having financial or demographic difficulties, for example, living far from the hospital or having a large household might impact the patients’ access to care, for example, by not having enough money for transportation. Furthermore, what factors contribute exactly to good disease management and blood sugar control among Rwandan individuals with T1 diabetes is still not completely clear. Therefore, all the information in the EMR system can be used to further investigate different factors that improve care and outcomes.

### Limitations

This study mainly focused on the needs related to features and content and focused less on more technical aspects, such as the data model, network architecture, and software type [8,12]. This limitation could be explained by the characteristics of the study participants, primarily consisting of HCPs and individuals with T1 diabetes. Including more software developers or IT experts might have contributed to the identification of other important themes. Another overlooked topic was the sustainability of the system, including budgeting, timeline, and maintenance of the system. Many IT projects fail due to a lack of continued funding or a good sustainability plan. It is important to consider how the system keeps operating, what will happen in case of staff turnover, and if and when updates are needed in the system. It is also important to consider integration with the national health information and management system, or at least how the systems can support each other instead of operating in parallel and creating double work for HCPs.

Moreover, research must occur concurrently as the pathophysiology of T1 diabetes is not fully understood. Over time, the EMR system will support data for clinical and epidemiological research. We previously investigated the association between HbA_1c_ level and the development of nephropathy among individuals with T1 diabetes in Rwanda, where it was concluded that more data are needed to know what exactly affects the blood glucose levels and the development of diabetes related complications [20].

The suggested EMR system is an early version, and we cannot be sure that we have identified all relevant data elements. Interviewing more individuals living with T1 diabetes or other HCPs from other hospitals could have given other insights. It is understood that updates and revisions will be needed. Nevertheless, it is a strength of this study that triangulation between data sources and methodologies was applied to obtain a comprehensive understanding of the needs and requirements in this specific context [15]. Future research is needed to verify the ToC and analyze whether the system can be used as intended and will lead to the expected improvements in clinical outcomes, as also suggested in the MRC framework.

As shown earlier, many aspects of our study were in accordance with previous research from other settings. Thus, the EMR system and the development process may be replicable in other LMICs and for disease areas other than T1 diabetes, but we recommend taking caution and considering context-specific customization. We are critical of the “one-size-fits-all” approach, but neither do we believe that we have to “reinvent the wheel” if someone wants to adapt an EMR system for better disease management.

### Conclusions

This study concludes that themes related to “features” and “content” are important to identify and consider when developing an EMR system for T1 diabetes management in Rwanda. The suggested EMR system is expected to improve data quality, optimize workflows, save more time for patients, improve clinical values, and ensure more patients are referred to treatment of complications in a timely manner. Hopefully, this will lead to a slower progression of chronic complications, more research in the area to further improve and optimize care, and eventually a long-term impact on improving health, quality of life, and reduced mortality rates among individuals with T1 diabetes.

## Abbreviations

EMR: electronic medical record
FGD: focus group discussion
HbA1c: glycated hemoglobin
HCP: health care professional
HIC: high-income country
LMIC: low- and middle-income country
RDA: Rwanda Diabetes Association
ToC: theory of change
T1 diabetes: type 1 diabetes
